# Awareness of prevention of teenage pregnancy amongst secondary school learners in Makhado municipality

**DOI:** 10.4102/phcfm.v8i2.967

**Published:** 2016-04-01

**Authors:** Giliana M. Maxwell, Makondelele Radzilani-Makatu, James F. Takalani

**Affiliations:** 1 Department of Psychology, University of Venda, South Africa

**Keywords:** Effectiveness, Sexuality Education, Teenage Pregnancy, Teenagers

## Abstract

**Background:**

Sexuality plays a very significant role in the lives of both boys and girls. It is, therefore, considered important for schools to recognise and accept sexuality as part of the development process of the child. Professor Kader Asmal (previous South African Minister of Education) suggested that the earlier the school begins to teach learners about sexuality, the better because they can be easily misled by their peers if proper guidance regarding their sexuality is not given.

**Aim:**

The current study was conducted to assess the awareness of teenagers on the prevention of teenage pregnancy (TP) in six secondary school learners situated in the Soutpansberg-West circuit, Makhado Municipality in Limpopo province.

**Setting:**

The study was conducted at six secondary schools situated in the Soutpansberg-West circuit, Makhado Municipality in Limpopo province in 2014.

**Methodology:**

A quantitative descriptive survey study was conducted where data were collected, using self-administered questionnaires, from 381 systematically sampled participants from six secondary schools situated in the Soutpansberg-West circuit, Makhado Municipality in Limpopo province. Data were analysed descriptively using the Statistical Package for the Social Sciences (SPSS) software, version 22.0. Necessary approval procedures and ethical clearance were obtained prior to data collection.

**Results:**

Ninety-four percent of participants agreed that TP can be prevented through abstaining from sex, whilst 65% of participants agreed that TP could be prevented by using contraceptives such as pills and injections. Eighty-three percent of participants agreed that TP could be prevented through the use of condoms. Seventy-four percent participants disagreed that bathing after sex prevents teenage pregnancies. Furthermore, 28% participants agreed that TP can be prevented by oral sex.

**Conclusion:**

The conclusion drawn was that learners are aware of the measures for preventing TP.

## Introduction

Children are sexualised human beings, and it is important for them to understand the sexual nature of their bodies.^1^ Sexuality education plays a very significant role in the lives of both boys and girls in the prevention of HIV and/or AIDS, sexually transmitted infections, as well as TP. It is, therefore, considered important for schools to recognise and accept sexuality education as part of the development process of the child. Professor Kader Asmal suggested that the earlier the school begins to teach children about sexuality, the better because they can be easily misled by their peers if proper guidance regarding their sexuality is not given.^1^

The Department of Education^2^ developed the sexual education programme (SEP) for implementation by the Department of Basic Education in 2001. Policy guidelines were established, and teachers were provided with workshops to prepare them to teach sexuality education programmes effectively. The examinable learning programme called Life Orientation, in which sexuality education forms part of the subject, was introduced to be taught in secondary schools in 2002.^2^

The aims and objectives of sexuality education prescribed by the Department of Education^1^ in the guidelines for teachers are, (1) to make young people like and respect themselves, (2) to help learners see sexuality as a natural and positive part of life, (3) to teach skills to make informed and responsible decisions, as well as (4) to explore different values and attitudes. Learners are further helped to act in accordance with their values, to understand, tolerate and respect their different sexual needs, orientations and values. They are further taught to protect themselves from exploitation and not to exploit others, and also how to use health services and how to find information they need.^1^ Numerous media reports have been questioning the effectiveness of this programme, and accusations that the schools or teachers were not doing enough or skirting their responsibilities have been made.^3^

Teenage pregnancies have doubled in the past years, despite a decade of spending on sexuality education.^3,4^ Van Eijk indicated that, despite the extensive attention given to adolescent sexuality and TP in the past 30 years, many teenagers were still falling pregnant.^4^ Panday et al. have emphasised that increased TP is more likely caused by lack of knowledge and information with regard to sexuality activities.^5^ Richter and Mlambo further indicated that TP is much encouraged by lack of access to sex education.^6^ According to Panday et al., the studies by the Medical Research Council in 2002 and 2008 both emphasised that misinformation was a causative factor of the high rate of TP.^5^

According to Panday et al., the South African National Youth Risk Behaviour conducted by the Medical Research Council in 2002 examined the prevalence of behaviour that places secondary school pupils at risk for diseases and ill health and discovered that a substantial number of young people are engaging in unprotected sex.^7^ The study also revealed that one in three teenagers had become pregnant before reaching the age of 20 years. It further concluded that 11% of pregnancy terminations were done by girls younger than 18 years of age. When the same study was conducted in 2008, it found out that 24.4% of the girls surveyed admitted to having been pregnant. The percentage was 5.3% more than when the study was conducted in 2002.^5^

The purpose of the study is to assess the awareness of learners about the prevention of TP through a sexuality education programme. The study intended to find out whether teenagers are aware of the different pregnancy preventative measures.

## Research methods and design

### Research design

A quantitative cross-sectional descriptive survey study was conducted to evaluate and describe the effectiveness of sexuality education (SE) for the reduction of teenage pregnancies (TP). The researcher conducted a cross-sectional descriptive design to provide an explicit description and to address the awareness of TP prevention by learners.^8^

### Study population

The total population for this study were Grade 11 and 12 secondary school learners at Sinthumule/Kutama area. Grade 11 and 12 learners were used as they had been taught SE since Grade 9, and they possess the characteristics required for the study to be productive.^9^ The researcher needed to target the population with the required characteristics, and which were representative of the desired population.^9,10^

### Sampling and sample size

Sampling refers to the selection procedure of people who possess the characteristics of the desired population for the study.^10^ The researcher used a random sampling method to select the schools used in the study as well as to select learners, and Slovin’s statistical formula was used to determine the sample size per school.

### Schools selection procedure

The researcher obtained the list of secondary schools from the Soutpansberg-West circuit offices. The area consists of 12 secondary schools, and the total population of Grade 11 and 12 learners was 678. Twelve pieces of paper with school names were placed in a bowl, and the bowl was shaken thoroughly before six secondary schools were picked from the bowl.

### Sample size calculation

Six randomly sampled secondary schools had a population of 451 in total. To determine the estimated sample size per school, the following Slovin statistical formula was used:

$$n=\frac{N}{1+N{\left(e\right)}^{2}}$$ [Eqn 1]

Where:

$$\begin{array}{l} n=\text{expected}/\text{estimated sample size}\\ N=\text{Total population per school}\\ e=\text{Expected error of response} \end{array}$$ [Eqn 2]

Researchers expected a response rate of 95% with only a 5% (*e* = 0.05) anticipated error margin. Slovin’s statistical formula was used to calculate the sample size in each school. From the sampled schools, the following sample sizes were found per school using Slovin’s statistical formula, as outlined on the table below. The sample sizes were calculated based on the researcher’s expected response rate of 95%, and the expected error margin of 5% (*e* = 0.05). Table 1 below indicates the sample size per school from six randomly selected secondary schools in the Soutpansberg-West circuit.

**TABLE 1:** Sample size per school.

School name	Sample size
1. Kutama Secondary School	79
2. Madaheni Secondary School	38
3. Maneledzi Secondary School	92
4. Sinthumule Secondary School	94
5. Swobani Secondary School	52
6. Tshirululuni Secondary School	26

**Total sample size**	**381**

*Source*: Authors own work

### Learners’ sampling procedure

After determining the sample size per school, learners were randomly sampled in the same manner as that for the schools. The process involved writing names of Grade 11 and 12 learners in each school and then placing them in a bowl. After shaking the bowl, participants were selected until the desired sample size was reached for each school.

### Data collection

A self-administered questionnaire was constructed following a literature review, and the content of Life Orientation Grade 10, 11 and 12 text books was used to collect data for this study.^9^ The questionnaire consisted of 16 questions with a three-point Likert-type scale rating: Agree, Not sure and Disagree. One school with 38 participants (10% of the sample) was used for pilot testing of the reliability of the questionnaire and the research methodology.^10^ The school used for this purpose was not included in the study, so as to avoid familiarity with the questionnaires.

Participants completed questionnaires anonymously and voluntarily after being reassured that the data collected would not be used to the detriment of all involved in the research project. Consent was sought from the parents of the learners sampled for the study in order to abide by the ethical code of conduct. Participants were given questionnaires to complete in the classroom at the same time in each school. The time allocated for their completion was 45 minutes. To avoid disturbance to normal teaching periods, data were collected in the afternoon during self-study time. Data collection was conducted in one secondary school per day in a two-week period. Three hundred and eighty-one questionnaires were administered and all 381 questionnaires were returned. The questionnaires were self-administered with the help of three trained research assistants. According to Maree, the advantage of this was that the researcher was able to gather information from all participants at the same time and to assist with issues in the questionnaires which were not clear to the participants.^10^

### Data analysis

Data analysis commenced after all questionnaires were collected from the six secondary schools. However, data was captured each day after data collection, i.e. data was captured in Microsoft Excel software the same day it was collected. After all the questionnaires were captured, the Excel data sheet was then imported to SPSS for analysis.

Questionnaires were analysed with the help of a statistician, using the SPSS V22.0 software program. The SPSS is ideal because it makes analysis quicker as the software recognises the location of the cases and variables after the data has been captured.^11^ It offers a great range of methods, graphs and charts. SPSS can take data from almost any type of file and use them to generate tabulated reports, charts, and plots of distributions and trends, descriptive statistics and complex statistical analysis.^12^ The data were then presented using descriptive statistics. Descriptive statistics are easily read and interpreted in tables, charts and frequencies format.^12^

## Ethical considerations

The research was conducted after approval was granted by the University of Venda, School of Health Sciences Committee as well as the University’s Higher Degrees and Ethics Committee (Project number: SHS/14/PSYCH/01/1508). Permission to gain entry to the research population was obtained from the Department of Education, Limpopo Province and the Soutpansberg-West circuit as well as the principals for the research to be conducted in schools.

The researcher ensured that participants knew about the purpose of the study by describing the aims of the research and procedures to be used.^9^ The researcher also provided an opportunity to answer questions by participants and their parents or guardians about the research process. The participants’ parents were informed that their children had a right to withdraw from the research at any stage. The participants’ parents were required to provide written consent that they allowed their children to participate in the research process, because the dominant age of learners at secondary schools is below 18 years.^13^ No personal identifying information was taken from the participants.^8,9,10^ The questionnaires were completed anonymously, and information received from the participants was kept confidential. In the study, no physical activities were given to the participants other than being required to complete and return the questionnaire within 45 minutes.^9^

## Results

### Demographic characteristics of the participation

The ages were distributed between 13 and 25 years. Participants between 13 and 15 years were 1% and those between 16 and 19 years were 68%. Thirty-one percent participants were between 20 and 25 years. Forty-three percent of participants were male, whilst 57% were female. Eighty-two percent of participants were Tshivenda speakers, 16% were Northern Sotho speakers, whilst only 2% of learners were Tsonga- speaking. From the study, 54% of participants were doing Grade 11, whilst 46% of participants were doing Grade 12.

Eighty-four percent of the participants were living with their parents, whilst 16% were not. Table 2 indicates that participants living with their mothers comprised 42%, whilst those living with their fathers were only 3%. The numbers of those living with both parents were slightly below those living with their mothers at only 38%, and 16% of the participants were not living with their biological parents.

**TABLE 2:** Demographic characteristics of the participation.

Biographic description	Frequency	Percentage
**Age**		
13–15 years	2	1
16–19 years	260	68
20–25 years	119	31
**Gender**		
Male	164	43
Female	217	57
**Home language**		
Tshivenda	312	82
Northern Sotho	63	16
Tsonga	6	2
**Grade**		
Grade 11	207	54
Grade 12	174	46
**Living with parents**		
Yes	319	84
No	62	16
**Category of parents**		
Both parents	146	38
Mother only	160	42
Father only	13	3
Not living with parents	62	17

*Source*: Authors own work

### Findings

The findings by this study show that learners are aware of the preventive measures of TP. Table 3 below shows that, out of 381 participants, 349 (94%) participants agreed that TP can be prevented through abstaining from sex. The results further revealed that 234 (65%) participants agreed that TP could be prevented by using contraceptives such as pills and needles.

**TABLE 3:** Distribution of participants’ responses regarding the awareness of learners on the prevention and reduction of teenage pregnancy (*N* = 381).

Teenage pregnancy can be prevented by	Response frequency and percentile					

	Agree		Not sure		Disagree	
		
	Frequency	%	Frequency	%	Frequency	%
1.1. Abstaining from sex	349	94	12	3	12	3
1.2. Using contraceptives (pills, needles etc.)	234	65	50	14	77	21
1.3. Using condoms	304	83	19	5	45	12
1.4. Bathing after sex	40	11	54	15	269	74
1.5. Oral sex	102	28	79	22	200	50
1.6. Sex whilst standing	24	7	52	14	291	79

*Source*: Authors own work

Furthermore, 304 (83%) participants agreed that TP could be prevented through the use of condoms. The results further show that 40 (11%) participants agreed that TP could be prevented by bathing after sex, whilst 269 (74%) participants disagreed that bathing after sex prevents TP. Furthermore, 102 (28%) participants agreed that TP can be prevented by oral sex. One hundred and eighty-four (50%) of participants disagreed that oral sex could prevent TP, whilst 79 (22%) participants were unsure.

Even though the findings show that the majority of participants were aware that oral sex cannot prevent TP, it is evident that teenagers still needed more information about the prevention of pregnancy. And, lastly, it is shown that 24 (7%) participants agreed with the statement that TP could result by having sex whilst standing. Fifty-two (14%) participants were unsure whether having sex when standing prevented TP, whilst 291 (79%) participants disagreed with the statement that having sex when standing could prevent pregnancy.

## Discussion of findings

The results revealed that teenagers were aware that TP could be prevented by properly using contraceptives, for example, pills, needles and condoms. This correlates with Myeza,^14^ who found that participants agreed that the proper use of contraceptives serves as the next best prevention measure crucial in TP. The majority of the participants seemed to understand the information in their text books, such as that in Gumede et al.,^15^ which described the measures for preventing TP. This high rate of agreement by learners that contraceptives prevent pregnancy meets one of the aims of SE that learners should be helped to develop an awareness of potential threats to their sexual safety and to learn skills for preventing, or coping with, such situations.^1^

The study determined that teenagers know that the proper use of condoms can prevent unexpected pregnancies. As indicated above, the study by Myeza^14^ shows that condoms and contraceptives were the second best preventive pregnancy methods, and learners seemed to be aware of this as the majority responded positively to the statement.

The study further revealed that teenagers were aware that bathing after sexual intercourse does not prevent pregnancy. However, there were some who were not sure if bathing after intercourse prevented pregnancy or not. The findings above indicate a need for on-going preventive programmes, as some of the participants were either not sure, or agreed, that bathing after sex prevented pregnancy. These findings are supported by Mchunu^16^ that, even though sexuality information is being communicated with learners effectively, there is still a need to enhance the teaching of SE, because some learners still lack information about TP prevention measures.

The results revealed that the majority of teenagers disagreed that oral sex prevented pregnancy. From the findings it is evident that almost half of the participants (50%) were not knowledgeable about whether oral sex is a preventive measure or not. According to their Grade 11 text book, oral sex has a zero chance of causing pregnancy.^15^ This view correlates with Panday et al. who emphasised that increased TP is more likely caused by lack of knowledge and information with regard to sexuality activities.^5^ Richter and Mlambo^6^ also indicated that TP is encouraged by the lack of access to sex education. According to Panday et al., the studies by the Medical Research Council in 2002 and 2008 emphasised that misinformation was a causative factor of the high rate of TP.^5^ From the study it was found that teenagers knew that pregnancy could not be prevented by having sexual intercourse whilst standing or in a standing position. From the findings it is evident that the majority of participants understood that sex whilst standing did not prevent pregnancy.^15^

## Recommendations

The following recommendations were made:

- Teachers need continuous trained on conducting SE and on encouraging learners to open up in discussing sexual-related issues.
- The Department of Education, in conjunction with other government stakeholders such as the Department of Health, needs to establish an awareness campaign with regard to the prevention of TP in rural areas such as Sinthumule/Kutama.
- SE should be integrated with other subjects rather than it only being part of the Life Orientation subject.

## Conclusion

The findings of the study indicate a beneficial role that the SE programme has played in alerting teenagers of the preventive measures for TP since its implementation in 1999. It is evident that, although there are some learners lacking knowledge in some of the sexual-related issues, to date, the majority are acquiring proper knowledge.

The conclusion drawn was that learners are aware of the preventive measures for TP.

## References

[1] South Africa. Department of Education. Protecting the right to innocence: Importance of sexuality education: Report of the protecting the right to innocence conference on sexuality education [homepage on the Internet]. c2001 [cited 2012 Oct 28]. Available from: http://www.dhet.gov.za/Reports%20Doc%20Library/Protecting%20the%20right%20to%20innocence%20The%20importance%20of%20Sexuality%20Education.pdf.

[2] South Africa. Department of Education. National Educational Act, 1996 [revised 1999]. Pretoria: Department of Education; 1999.

[3] Naidoo M An evaluation of sexuality education programme implemented in South African schools [homepage on the Internet; unpublished PhD thesis]. South Africa: University of Zululand; 2006 [cited 2012 Oct 23]. Available from: http://uzspace.uzulu.ac.za/bitstream/handle/10530/246/An+evaluation+of+th+sexuality+education+programme+being+implemented+in+South+Africans+schools+-+M.pdf?sequence=1.

[4] Van Eijk RT Factors contributing to teenage pregnancies in Rarotonga [homepage on the Internet]. Avarua: United Nations Population Fund (UNFPA); 2007 [cited 2012 Sep 04]. Available from: http://countryoffice.unfpa.org/pacific/drive/TeenPregnancies_Cooks.pdf.

[5] Panday S, Makiwane M, Ronchad C, Letsoalo T Teenage pregnancy in South Africa: With a specific focus on school-going learners. Commissioned by UNICEF [homepage on the Internet]. 2009 [cited 2012 Aug 17]. Available from: http://www.education.gov.za/LinkClick.aspx?fileticket=uIqj%2BsyyccM%3D&.

[6] Richter MS, Mlambo GT Perceptions of rural teenagers on teenage pregnancy. Health SA Gesondheid [homepage on the Internet]. 2005;10(2):61–69. [cited 2014 March 20]. Available from: http://www.ajol.info/index.php/hsa/article/view/10311.

[7] Love Life Report: A National survey of South African teenagers [homepage on the Internet]. c2008 [cited 2012 Oct 28]. Available from: http://www.lovelife.org.za/corporate/files/4713/3848/3460/South_African_National_HIV_prevalence_incidence_behaviour_and_communication_survey_2008_A_turning__tide_among_teenagers.pdf.

[8] Burns N, Grove SK Understanding nursing research. 3rd ed Philadelphia, PA: Saunders; 2005.

[9] De Vos AS, Strydom H, Fouche CB, Delport CSL A research at grassroots: For the social sciences and human service professionals. 4th ed Pretoria: Van Schaik; 2011.

[10] Maree K First steps in research. Pretoria: Van Schaik; 2007.

[11] David H Benefits of SPSS [homepage on the Internet]. c2012 [cited 2013 Feb 4]. Available from: http://benefitof.net/benefits-of-spss.

[12] Jaggi S, Batra PK SPSS: An overview. Design of experiment application [homepage on the Internet]. c2013 [cited 2013 Feb 04]. Available from: http://www.iasri.res.in/iasriwebsite/DESIGNOFEXPAPPLICATION/Electronic-Book/Module%201/6SPSS-overview.pdf.

[13] Babbie E, Mouton J The practice of social research. Cape Town: Oxford University Press; 2009.

[14] Myeza NP Attitudes of high school learners towards sexuality education in Zululand [homepage on the Internet; unpublished dissertation]. South Africa: University of Zululand; 2008 [cited 2014 July 13]. Available from: http://uzspace.uzulu.ac.za/bitstream/handle/10530/125/Attitudes+of+high+school+learners+towards+Sexuality+Education+-+NP+Myeza.pdf?sequence=1.

[15] Gumede H, Martinuzzi PT, Moore LM, et al Focus on life orientation: Grade 11. Cape Town: Maskew Miller Longman; 2006.

[16] Mchunu NJ Teachers’ perceptions of the teaching of sexuality education in secondary schools in Pinetown district [homepage on the Internet; unpublished dissertation]. University of Zululand: South Africa; 2007 [cited 2014 Jul 13]. Available from: http://researchspace.ukzn.ac.za/xmlui/bitstream/handle/10413/1351/Mchunu_NJ_2007.pdf?sequence=1.

